# Optimization of CRISPR/Cas12 tools for plant genome editing

**DOI:** 10.1093/plphys/kiaf345

**Published:** 2025-08-01

**Authors:** Sara Selma

**Affiliations:** Assistant Features Editor, Plant Physiology, American Society of Plant Biologists; VIB Center for Plant Systems Biology, Ghent 9052, Belgium

Since the advent of CRISPR systems as gene-editing tools, the ability to introduce targeted mutations has increased exponentially. CRISPR systems are relatively simple to use, requiring only a Cas protein (CRISPR associated), which is an endonuclease, and a small guide RNA containing a sequence homologous to the target genomic region. Additionally, the CRISPR system requires a PAM sequence (protospacer adjacent motif), a short DNA sequence adjacent to the target region, which allows recognition by the Cas protein. In the case of Cas9, the typical sequence should be NGG, but it can be optimized to change the PAM requirements and thus its specificity (Gan and Ling 2022). All these characteristics, combined with its versatility, have led to the rapid and widespread expansion of CRISPR globally.

However, since the first reports of Cas9 from *Streptococcus pyogenes* as a plant-editing tool, CRISPR systems have undergone a revolution in terms of the types of Cas proteins, their bacterial source, and optimization. One of the emerging alternative tools to Cas9 is the CRISPR system based on Cas12a, formerly CPF1. This endonuclease belongs to the Class 2 Type V-A CRISPR/Cas system. It presents a series of characteristics that could be advantageous, such as a smaller Cas protein size, the recognition of an alternative PAM (TTTV), the generation of sticky end cuts (instead of the blunt ends generated with Cas9), and greater simplicity and self-processing of the small guide RNA (i.e. CRISPR RNA [crRNA]), which allows “multiplexing” or the crRNA tandem construction (Zhang et al. 2021).

The types of Cas12a characterized to date come from different bacterial origins, such as FnCas12a from *Francisella novicida*; AsCas12a, whose origin is *Acidaminococcus* sp.; or LbCas12a from *Lachnospiraceae bacterium*. The last of which has been successfully employed in different studies of plant genome editing (Malzahn et al. 2019). However, its use is not yet completely popular due to the limitations that this system still presents, such as temperature sensitivity and suboptimal cleavage activity (Bandyopadhyay et al. 2020). For this reason, its optimization represents a fascinating challenge, as it can expand the toolbox available in plant biology research and its applications.

Previous studies showed optimizations of the LbCas12a system for plant species (Zhang et al. 2021). For example, Xin et al. (2024) showed the ttLbCas12a Ultra V2 (ttLbUV2), an ultraoptimized variant of LbCas12a, especially in *Arabidopsis thaliana*. ttLbUV2 includes 2 key mutations: D156R, which improves tolerance to operation at lower temperatures, and E795L, which increases catalytic activity. Additionally, ttLbUV2 adds an optimized nuclear localization signal (NLS) and codon usage, which greatly enhance its editing efficiency.

However, the most common question that we might ask ourselves when designing an experiment that requires efficient genetic editing in plants is “Which of the CRISPR options should I choose?” To help us with the choice, Xin et al. (2025) performed an extensive evaluation of different LbCas12a variants but also optimized 2 Cas12i3 variants (Class 2 Type V-I CRISPR/Cas) and 2 AsCas12f variants (Class 2 Type V-F CRISPR/Cas). Cas12i3 shows high flexibility in PAM preference (TTN vs. TTTV) but a smaller protein size. AsCas12f1 is one of the smallest Cas nucleases and recognizes YTTN or NTTR PAMs. Additionally, the authors addressed another outstanding question regarding ttLbUV2: which of the 2 optimization strategies—varying NLS sequences or optimizing codon usage—is more effective at increasing editing efficiency?

As a first approach, the editing capacity of ttLbUV2 was evaluated across 18 targets, showing high editing efficiency and minimal target bias. Twelve belong to different positions of the gene *GL1*, showing editing efficiencies that ranged from 20.8% to 99.1% for homozygous or biallelic mutations in T1 transgenic plants. The next evaluation includes 3 pairs of homologous targets for simultaneous mutations in CHLI1 and CHLI2, using a single crRNA to target both genes. This crRNA sequence included distal mismatches from the PAM sequence to evaluate the mismatch tolerance of ttLbUV2. The obtained efficiencies were 85.4%, 95.3%, and 99.3% of crRNA 1&2-1, 1&2-2, and 1&2-3, respectively. These results demonstrate high editing efficiency combined with tolerance to 1 or 2 PAM-distal mismatches. Finally, the position of the crRNAs inside the tandem was assessed by targeting the genes *TRY* and *CPC* using 2 vectors, in which the order of the crRNAs was swapped. The results confirmed that the order of the crRNAs does not affect the editing efficiency, with 97.8% for the T&C crRNA array and 96.1% for the C&T crRNA array. No off-target mutations were detected in computationally predicted sites for all the targets evaluated, confirming the specificity of ttLbUV2. Additionally, in the T2 T-DNA–free plants, all observed mutations were heritable.

Later, to elucidate whether the critical factor for the improvement of the editing efficiency is the NLS optimization or the codon usage, the authors compared the efficiency of the ttLbCas12a Ultra variants: the ttLbUV0, the original variant with no NLS optimization; the ttLbUV1, which shares the same protein sequence as ttLbUV2, including NLS, but retains the original codon usage from ttLbUV0; and, of course, the ttLbUV2. The editing efficiencies targeting the genes *ECA3-1*, *GL2*, and *TT4* showed the lower efficiency of ttLbUV0. Still, no remarkable differences were found between ttLbUV1 and ttLbUV2, pointing to the determinant effect of the NLS over the codon usage.

The authors developed 4 additional variants: LbCas12a-RRV (RRV), LbCas12a-RRVL (RRVL), hyperCas12a (hyper), and hyperCas12a Ultra (hyperU; Guo et al. 2022; Zhang et al. 2023), with the same NLS and codon usage but carrying different amino acid mutations in their sequence. The editing efficiencies of these variants targeting *ECA3-1*, *GL2*, and *TT4* showed that although RRVL exhibits the highest editing efficiency, the minimal differences in terms of editing efficiency are not enough to switch out of the ttLbUV2 variant.

Finally, to increase the CRISPR toolbox for plant genome editing, Cas12i3 and AsCas12f were assessed as potential candidates. Two Cas12i3 variants and 2 AsCas12f variants were designed: Cas12i3V1 and Cas12i3V2, with S7R, D233R, D267R, N369R, and S433R mutations, and AsCas12f-YHAM and AsCas12f-HKRA, with an optimized small guide RNA scaffold. The variants include identical NLS sequences and were codon optimized for dicot plants except for Cas12i3V1, which was codon optimized for monocots. Cas12i3 variants showed relatively high editing efficiency at 4 of 6 tested targets, with Cas12i3V1 unexpectedly outperforming Cas12i3V2, suggesting that factors beyond codon optimization affect efficiency. Unfortunately, AsCas12f variants showed poor or no detectable editing, with only weak chimeric mutations observed from AsCas12f-YHAM.

In sum, Xin et al. performed a comprehensive evaluation of editing efficiency in different LbCas12a variants, achieving high percentages of editing with the variants RRVL and ttLbUV2. This work concludes that the design of the NLS in the CRISPR systems is a determinant for optimizing their efficiency, rather than the codon usage. Finally, the Cas12i3V1 was presented as a promising genome-editing tool for *Arabidopsis*, expanding the toolbox of editing tools. Unfortunately, AsCas12f variants require further optimization for effective use in plants. The development of new CRISPR tools offers new opportunities for more efficient and complex genetic editing, expanding the range of editable plant species and offering new solutions for agricultural biotechnology.

## Data Availability

No new data were generated or analysed in support of this.
